# *Mycobacterium tuberculosis* Complex and HIV Co-Infection among Extrapulmonary Tuberculosis Suspected Cases at the University of Gondar Hospital, Northwestern Ethiopia

**DOI:** 10.1371/journal.pone.0150646

**Published:** 2016-03-07

**Authors:** Alemu Fanosie, Baye Gelaw, Belay Tessema, Wogahta Tesfay, Aschalew Admasu, Gashaw Yitayew

**Affiliations:** 1 School of Biomedical and Laboratory Sciences, College of Medicine and Health Sciences (CMHS), The University of Gondar (UOG), P.O. box 196, Gondar, Ethiopia; 2 School of Medicine, College of Medicine and Health Sciences (CMHS), The University of Gondar (UOG), P.O. box 196, Gondar, Ethiopia; 3 Bahir Dar Regional Health and Research Laboratory Center, Bahir Dar, Ethiopia; University of Cape Town, SOUTH AFRICA

## Abstract

**Background:**

Extrapulmonary Tuberculosis (EPTB) and Human Immunodeficiency Virus (HIV) infection are interrelated as a result of immune depression. The aim of this study was to determine the prevalence of *Mycobacterium tuberculosis* complex isolates and the burden of HIV co-infection among EPTB suspected patients.

**Method:**

An institution based cross-sectional study was conducted among EPTB suspected patients at the University of Gondar Hospital. Socio-demographic characteristics and other clinical data were collected using a pretested questionnaire. GeneXpert MTB/RIF assay was performed to diagnosis *Mycobacterium tuberculosis* complex and Rifampicin resistance. All samples were also investigated by cytology and culture. The HIV statuses of all patients were screened initially by KHB, and all positive cases were further re-tested by STAT-pack. Data was analyzed using SPSS version 20 computer software and a P-value of < 0.05 was taken as statistically significant.

**Results:**

A total of 141 extrapulmonary suspected patients were enrolled in this study. The overall prevalence of culture confirmed extrapulmonary tuberculosis infection was 29.8%, but the GeneXpert result showed a 26.2% prevalence of *Mycobacterium tuberculosis* complex infection. The 78.4% prevalence of extrapulmonary tuberculosis infection was found to be higher among the adult population. The prevalence of HIV infection among EPTB suspected patients was 14.1%, while it was 32.4% among GeneXpert-confirmed extrapulmonary TB cases (12/37). Tuberculosis lymphadenitis was the predominant (78.4%) type of EPTB infection followed by tuberculosis cold abscess (10.7%). Adult hood, previous history of contact with known pulmonary tuberculosis patients, and HIV co-infection showed a statistically significant association with extrapulmonary tuberculosis infection (P<0.013).

**Conclusion:**

The prevalence of culture confirmed-EPTB infection was high, and a higher EPTB-HIV co-infection was also observed.

## Introduction

Tuberculosis is predominantly associated with lung diseases, but it can also affect other parts of the body, extrapulmonary tuberculosis (EPTB). Globally, there were 8.6 million new TB cases in 2012, and an estimated 1 million people developed EPTB [1]. In Africa, the proportion of EPTB infection was reported as 17.7% by the year 2013 [2]. In Ethiopia, a 32.5% EPTB proportion was reported by the year 2012 among the total TB cases [3].

Extrapulmonary tuberculosis most commonly occur at sites, such as lymph node, pleura, bone and joints, central nervous system, ocular, pancreatic and genitourinary tract [4,5]. In immune-competent hosts, *Mycobacterium tuberculosis* complex dissemination to other tissues is usually controlled. In immune-compromised patients however, the tubercle bacilli may disseminate to different parts of the human body [6, 7].

Before the beginning of the Human Immunodeficiency Virus (HIV) epidemic, about 85% of the reported tuberculosis cases were limited to the lungs [8]. This distribution has been substantially different since the emergence of HIV [9], because the frequency of the extrapulmonary TB tends to increase if the immune function is compromised [10]. Previous report showed that, in countries with HIV epidemic, there were dramatic increases in extrapulmonary TB cases and deaths because of immunity deteriorations [11]. Mortality in HIV-associated extrapulmonary tuberculosis was high because of a combination of higher stage HIV disease-related opportunistic infections and delays in the diagnosis and treatment of the disease [12]. The deadly TB-HIV synergy and the occurrence of the multidrug-resistant *M*. *tuberculosis* (MDR-TB) have further complicated tuberculosis control and increased the development of active TB [13, 14]. In 2013, there were an estimated 9 million incidental cases of tuberculosis in the world, out of which 1.1 million were HIV positive. This TB/HIV co- infection and related deaths are high in African region. In Ethiopia, the prevalence of HIV co-infection among TB patients was reported as 5.9 per 100,000 [15].

According to reports, the incidence of EPTB has been increasing among TB patients across Ethiopia since the 1990’s [16]. However, Iwnetu et al disagreed with the report on the ground that the increase might be observed due to a simple over diagnosis and stated that up to 15% of all tuberculosis lymphadenitis (TBLP) cases could be wrongly diagnosed [17]. Although reports document that cytology has a lower specificity as a method of diagnosis of EPTB infection, it is most frequently used for the purpose in Ethiopia. The chief difficulty with extrapulmonary specimens is that they yield very few bacilli which are consequently associated with a low sensitivity of acid-fast bacillus [18]. The prevalence of EPTB varies across studies from 15 to 50% and depends on the region, the ethnic group studied, and HIV co-infection rates [19]. In Gondar, studies done to evaluate the magnitude of this form of tuberculosis (EPTB), the various sites involved, and their association with HIV infection have been few so far. Therefore, this study was conducted to determine the prevalence of *Mycobacterium tuberculosis* complex and HIV co-infection among extrapulmonary tuberculosis suspected patients at the University of Gondar Hospital.

## Materials and Methods

### Study design, area and period

An institution based cross sectional study was conducted at the University of Gondar teaching Hospital. Gondar is located in the northwestern part of Ethiopia 739 km from the capital city, Addis Ababa. It is at latitude and longitude 12°36′N 37°28′E, with an elevation of 2133 m above sea level. According to figures from the Central Statistical Agency 2008, Gondar has an estimated total population of 231,977. The University of Gondar Hospital, a referral Hospital for northwest Ethiopia with more than 400 beds is serving a population of about 5 million. One of the major investigation services given at the Hospital is the diagnosis and treatment of extrapulmonary tuberculosis infection. The diagnosis service is mainly accomplished at the Department of Pathology, where a number of samples are collected and investigated. This study was conducted between March to April 2015.

### Source and study populations

All extrapulmonary tuberculosis suspected patients seeking health services at the University of Gondar Hospital were used as source population for this study. The extrapulmonary tuberculosis suspected patients for whom samples were collected from different body sites and submitted to the Pathology Laboratory of the University of Gondar were used as the study population.

### Inclusion and Exclusion criteria

Extrapulmonary tuberculosis suspected patients willing to participate in the study were included, but previously confirmed pulmonary tuberculosis (PTB) cases and known extrapulmonary tuberculosis patients who were on anti-TB treatment were excluded from the study.

### Variables of the study

The prevalence of *Mycobacterium tuberculosis* complex/HIV co-infection among EPTB suspected cases was used as the dependent variable, whereas age, sex, marital status, family size, housing condition, monthly average income, smoking status, residence, previous history of TB infection, previous treatment for TB, contact with TB patients, alcohol consumption, and history of chronic illness were used as independent variables.

### Sample size

The sample size was determined using the following single population proportion formula: N = z^2^ p (1-p)/ w^2^, where N = the number of EPTB suspected patients; Z = Standard normal distribution value at 95% CI which is 1.96; P = the prevalence of extrapulmonary tuberculosis infection = 9.9% (24); W = the margin of error taken as 5%. Accordingly, a total of 141 samples were collected from different body sites of EPTB suspected patients.

### Sampling technique

Samples were collected from the extrapulmonary tuberculosis suspected patients who came to the Pathology Laboratory and inpatients admitted to different wards at the University of Gondar Hospital consecutively until the required number of patients offered the specimens.

### Socio-demographic and clinical data

Socio-demographic information’s included in this study comprised age, sex, marital status, family size, housing condition, monthly average income, smoking status, and residence. In addition, previous history of TB infection, previous treatment for TB, contact with TB patients, alcohol consumption, and history of chronic illness were also investigated. Clinical information on each patient was collected after investigation by the same questionnaire. The questionnaire was prepared in English and translated to the local language (Amharic), after its completeness and validity was pretested at a place different from the study area, Gondar Polyclinic.

### Sample collection and processing

Fine needle aspirate (FNA) samples were collected by a pathologist, while other body fluid samples were collected by medical doctors during patient investigations. All the collected samples were delivered to the Pathology Laboratory for cytological examination, and the left over samples were used for this study. The HIV status of the participants was taken from the Providing Initiative Counseling and Testing (PICT) Clinic of the Hospital. Smears from FNA samples were prepared by depositing a drop of the aspirate and distributing it on a microscope slide. All body fluid samples were centrifuged at 3000 rpm for 15 minutes. The supernatant was discarded and the pellet was divided in to two parts. One-third of the sediment was smeared on a microscope slide for cytological investigation and the other two-third was re-suspended with sterile phosphate buffer saline solution (PBS) for GeneXpert MTB/RIF assay and culture. Samples from non-sterile sites such as a superficial infection of the skin and open wounds were decontaminated with 4% NaOH [20].

### Laboratory investigations

#### GeneXpert MTB/RIF assay

The assay machine had a single use multi-chamber plastic cartridge with lyophilized reagents and buffers for sample processing, amplification, and detection [21]. Clinical samples were manually transferred to the cartridge which was loaded into the GeneXpert instrument and subsequently processed automatically at the University of Gondar Hospital Laboratory. The sample was processed by diluting it in a 2:1 ratio with a sample reagent (SR) buffer. The result was presented in a text format by using a computer software [22].

#### Culture

All samples were subjected to culture at the Bahir Dar Regional Health and Research Laboratory Center, Bahir Dar, Ethiopia. Half ml of each sample was inoculated on two Lowenstein-Jensen (LJ) slops using sterile Pasteur pipette and incubated at 37°C in a slant position for 1 week. After 1 week, tubes were incubated in a standing position and caps were tightened to minimize evaporation and drying of the media. Then, colony formation was checked every week, preferably twice within the first week. *M*. *Tuberculosis* complex colonies were well- developed within 3–6 weeks, and cultures were kept for up to 8 weeks before being reported as negative.

#### Capilla test

Samples that were negative by the GeneXpert procedure but positive by culture were subjected to Capilla test. This is a rapid test for the identification of *Mycobacterium tuberculosis* complex. One hundred μl of the diluted bacteria was taken and placed on the area of the test plate of the Capilla slide. After 15 minutes of incubation, the result was displayed on the Capilla slide and graded as positive or negative for *Mycobacterium tuberculosis* complex.

#### Drug susceptibility test

The drug susceptibility pattern of the *Mycobacterium tuberculosis* complex was determined using the Line Probe Assay (LPA) test. The test is based on the DNA-strip technology and permits the molecular genetics identification of the *Mycobacterium tuberculosis* complex and its resistance to rifampicin and/or isoniazid. The manufacturer’s instruction demonstrated that the identification of rifampicin resistance is enabled by the detection of the most significant mutations of the rpoB gene (coding for the β-sub-unit of the RNA polymerase). For the detection of a high level isoniazid resistance, the katG gene (coding for the catalase peroxidase) was examined, and for the detection of a low level isoniazid resistance the promoter region of the inhA gene (coding for the NADH enoyl ACP reductase) was examined. Line probe Assay was accomplished following the manufacturer’s instructions (GenoType MTBDRplus, Hain Life science GmbH, 2009, Germany).

#### Cytological investigation

Smears from different body fluids were prepared, air dried, and stained with Wright stain solution. All the stained slides were examined by a senior pathologist. The presence of epitheloid cell granuloma with or without multinucleated giant cells was used as an evidence for extrapulmonary tuberculosis infection from FNA preparations. Granulomatous findings with or without caseous necrosis or degenerate caseous necrosis and liquefied necrotic material with marked degenerating and viable inflammatory cell infiltration without epitheloid granuloma were also considered as suggestive for extrapulmonary tuberculosis infection. In the case of body fluids, exudative effusion with lymphocyte predominance was suggestive of extrapulmonary tuberculosis infection [23].

#### Human immunodeficiency virus test (HIV-test)

The test results were collected from the Providing Initiative Testing and Counseling (PICT) Clinic of the Hospital. To detect HIV infection, the anti-HIV antibody test was used and performed following the manufacturer’s instructions. Moreover, the national HIV test algorithm for Ethiopia was followed. The algorithm describes the use of KHB for initial screening, and all KHB positive samples were re-tested with STAT-PACK for confirmation [24]. In this study, no discordant result was observed between KHB and STAT-PACK test results.

### Quality control

The reliability of the study findings were guaranteed by implementing quality control measures throughout the whole processes of the laboratory work. GeneXpert MTB/RIF assay has its own internal quality control system which was used during the investigation process. The quality or sterility of the prepared LJ culture media was assured by incubating 10% of the batch of the prepared culture media at 37°C for 48 hours. The performance of LJ culture media was determined using known H37RV *Mycobacterium tuberculosis* strain and non-tuberculosis mycobacteria (NTM). Line probe assay reagent quality was guaranteed by observing for the presence of any color change or precipitation, and the manufacturer’s instructions were strictly followed. Moreover, the quality of line probe assay procedure was maintained using H37RV *Mycobacterium tuberculosis* strain. The quality of Wright’s stain solution was assured by using previously tested smears. The questionnaire was pre-tested on 10 extrapulmonary tuberculosis suspected patients at the Gondar Polyclinic for its clarity and appropriateness, and the data collected was checked immediately for its consistency and accuracy.

### Data management and analysis

Data was entered and analyzed using the SPSS version 20 statistical computer software. Descriptive analysis was used to determine demographic and clinical characteristics and the prevalence of *Mycobacterium tuberculosis* complex and/or HIV co-infection. The association between the characteristics and the *Mycobacterium tuberculosis* complex and/or HIV co-infection was first analyzed by a series of bivariate analyses. Then, to control for the possible confounding effects of the different variables, the multivariate analysis was used. In both analyses, the association was expressed in odds ratios (OR) and 95% confidence intervals (CI). All tests of the multivariate analysis with a P-value of <0.05 were considered statistically significant.

### Ethical considerations

Ethical approval was obtained from an Ethical Review Committee organized by the School of Biomedical and Laboratory Sciences which was mandated by the College of Medicine and Health Sciences, the University of Gondar with reference number SBMLS/927/07, dated the 30^th^ of January 2015. A permission and support letter was also obtained from the University of Gondar Hospital, and both verbal and written consent was obtained from each participant. All consent procedures were approved by the Ethical Approval Committee of the School of Biomedical and Laboratory Sciences, College of Medicine and Health sciences, University of Gondar. In the case of children, both verbal and written consents were received from guardians and were approved by the Ethical Review Committee. To maintain the confidentiality of participant information, names and other identifiers were left out in the questionnaire, and interviews were carried out individually. All samples needed for diagnostic purposes were collected from patients by medical doctors, whereas only the left over samples were used by this study. Any positive findings were communicated to study participants, while GeneXpert positive study participants and MDR TB cases were referred to TB Clinic and got treatment according to the national guide line.

## Results

A total of 141 EPTB suspected samples were collected from different body sites of patients. The proportion of lymphnode aspirate samples was 61.7%, pleural fluid 12.8%, cold abscess 8.5%, skin lesions 6.4%, ascetic fluid 5%, genitourinary tract samples (testicular aspirate) 2.1%, peritoneal fluid 2.1%, and each pericardial and synovial fluid accounted for 0.7%.

### Socio-demographic characteristics of patients

More than half of the study participants (52.5%; 74/141) were male and 47.5% (67/141) were female. The mean age was 30.4 years (± SD, 18.9 year). Pediatric patients accounted for 20.6% (29/141) and adults for 79.4% (112/141). Marital status data showed that 39% were married, 25.5% single, 9.2% divorced and 5.7% widowed. About two-thirds (67.4%) of the study participants were living in rural areas, and 34% had a monthly income of less than 400 Ethiopian birr (equivalent to 20 dollars) “Table 1”. The majorities (73.8%) of the EPTB suspects lived in single rooms and 43.3% in rooms with no windows.

**Table 1:** Socio-demographic characteristics and prevalence of GeneXpert confirmed extrapulmonary tuberculosis infection at the University of Gondar Hospital (N = 141), February to April 2015.

Characteristics		Extra pulmonary Tuberculosis		
		GneXpert Positive N^o^ (%)	GneXpert Negative N^o^ (%)	Total N^o^ (%)
Sex	Male	20 (54.5%)	54 (52%)	74 (52.5%)
	Female	17 (45.5%)	50 (48%)	67 (47.50%)
Age in years	0–14	3 (8%)	26 (25%)	29 (20.6%)
	15–29	19 (51.4%)	31 (30%)	50 (35.5%)
	30–44	10 (27%)	16 (15.4%)	26 (18.4%)
	45–59	4 (11.8%)	20 (19%)	24 (17%)
	≥60	1 (2.7%)	11 (10.6%)	12 (8.5%)
Marital status	Single	13 (35.1%)	23 (22.2%)	36 (32.1%)
	Married	14 (38%)	41 (39.4%)	55 (49.1%)
	Divorced	6 (16.2%)	7 (6.7%)	13 (11.6%)
	Widowed	1 (2.7%)	7 (6.7%)	8 (7.2%)
Residence	Urban	10 (27%)	36 (34.6%)	46 (32.6%)
	Rural	27 (73%)	68 (65.4%)	95 (67.4%)
Family Size (Person)	< 4	21 (56.8%)	37 (35.8%)	58 (41.2%)
	4–7	9 (24.3%)	48 (46.2%)	57 (40.4%)
	>7	7 (18.9%)	19 (18%)	26 (18.4%)
No of Rooms	One	33 (89.2%)	71 (68.3%)	104 (73.8%)
	Two	4 (10.8%)	17 (16.3%)	21 (14.9%)
	Three	0	9 (8.7%)	9 (6.3%)
	Four	0	7 (6.7%)	7 (5%)

### Prevalence of GeneXpert and culture confirmed *Mycobacterium tuberculosis* complex infection among EPTB suspected cases

The GeneXpert MTB/RIF assay result showed that 37 out of 141 (26.2%) clinically suspected EPTB patients were positive for *Mycobacterium tuberculosis* complex. The prevalence of GeneXpert confirmed *Mycobacterium tuberculosis* complex infection among male and female was 27% (20/74) and 25.4% (17/67), respectively. Thirty-three out of the 37 GeneXpert confirmed EPTB cases (89.2%) were living in rooms that had no windows, and 56.8% (21/37) had families more than 4 members. On the other hand, 70.3% had history of contact with known pulmonary tuberculosis patients, and drank alcohol “Table 2”. All of the 141 samples were subjected to culture and the prevalence of the culture confirmed EPTB infection was 29.8%. Thirty-five out of the 37 GeneXpert positive samples were positive for *Mycobacterium tuberculosis* complex by culture. The sensitivity of the culture method was 89.4%, and that of GeneXpert 87.5%. Taking culture as gold standard, the specificity of the GeneXpert method was 95.2%. All culture positive but GeneXpert negative samples were positive for *Mycobacterium tuberculosis* complex by the Capilla test. Both GeneXpert and Line probe assay drug susceptibility tests showed only one *Mycobacterium tuberculosis* complex isolate showed multi-drug resistance.

**Table 2:** Comparison of GeneXpert test results with cytology microscopy for the diagnosis of extrapulmonary tuberculosis infection.

Sample type	GeneXpert result		Cytology result No		Total No (%)
	MTB No (%)	Not MTB No (%)	T No (%)	Not TB No (%)	
Lymph node aspirate	29 (33.3%)	58 (66.7%)	50 (57.5%)	37 (42.5%)	87 (61.7%)
Pleural fluid	1 (5.6%)	17 (94.4%)	7 (38.9%)	11 (61.1%)	18 (12.8%)
Ascetic fluid	0	7 (100%)	4 (57.1%)	3 (42.9%)	7 (5%)
Peritoneal fluid	0	3 (100%)	0	3 (100%)	3 (2.1%)
Pericardial fluid	0	1 (100%)	0	1 (100%)	1 (0.7%)
Genitourinary sample	1 (33%)	2 (66.7%)	1 (33.3%)	2 (66.7%)	3 (2.1%)
Pus	4 (33.3%)	8 (66.7%)	7 (58.3%)	5 (41.7%)	12 (8.5%)
Joint (synovial) fluid	0	1(100%)	0	1 (100%)	1 (0.7%)
Skin	2 (22.2%)	7 (77.8%)	6 (66.7%)	3 (33.3%)	9 (6.4%)

### Comparison of cytology, GeneXpert and culture results

The cytology result showed that 53.2% (75/141) of the samples had cellular appearance suggestive of tuberculosis infection. Taking culture results as true positives, the specificity of cytology was 75%. Moreover, 66.7% of the lymph nodes, 14.7% of body fluids, 9.3% of cold abscess, 8% of skin, and 1.3% of testicular samples were positive by cytology. However, the GeneXpert result revealed that only 36 out of the 75 samples were confirmed positive for *Mycobacterium tuberculosis* complex infection. Higher discordant results between cytology and GeneXpert assay were observed on pleural fluid samples that out of the seven cytology positive samples only one was positive for *Mycobacterium tuberculosis* complex infection by GenXpert assay. Nevertheless, concordant results between cytology microscopy and GenXpert were observed among samples collected from genitourinary tract, peritoneal fluid, pericardial fluid and joint fluids “Table 2”. Cohen’s Kappa was computed to evaluate the agreement between GeneXpert and cytological examinations. The result showed that the test agreement of the two methods was 0.45 indicating that these two methods do not provide similar results on the diagnosis of EPTB infection. The cytology and GenXpert results were also compared with the result of culture. Accordingly, the cytology test agreement with culture was 0.433 and the GeneXpert test result agreement with culture was 0.842.

### *Mycobacterium tuberculosis* complex and HIV co-infection

In this study, the HIV status of 135 patients was determined by a rapid HIV test method. The prevalence of HIV infection among the extrapulmonary suspected patients was 14.1% (19/135). On the other hand, the prevalence of HIV infection among GeneXpert confirmed extrapulmonary TB cases was 32.4% (12/37). Among HIV positive patients, the prevalence of *Mycobacterium tuberculosis* complex infection was 63.2% (12 of 19). Moreover, there was a statistically significant association between *Mycobacterium tuberculosis* complex and HIV infection (P<0.001). This association existed when it was adjusted by the multivariate logistic regression analysis for age, sex, residence and other clinical and behavioral covariates. Among the 42 culture positive TB cases, 11 were HIV positive, 30 HIV negative, and the HIV status of one patient was unknown. Among patients that had TB-HIV co-infection, the highest co-infection was seen in male patients (75%, 9/12) and in the age group of 15–29 years (66.7%, 8/12). A higher *Mycobacterium tuberculosis* complex/HIV co-infection (66.7%) was observed among unmarried rural dwellers (58.3%, 7/12) “Table 3”.

**Table 3:** Extra pulmonary tuberculosis and HIV co-infection among GeneXpert confirmed extrapulmonary tuberculosis patients at University of Gondar Hospital.

Characteristics		GeneXpert confirmed Extra pulmonary Tuberculosis		Total No (%)
		HIV positive No (%)	HIV Negative No (%)	
Sex	Male	9 (45%)	11 (55%)	20 (55.6%)
	Female	3 (18.8%)	13 (81.2%)	16 (44.4%)
Age (years)	0–14	0	3 (100%)	3 (8.3%)
	15–29	8 (44.4%)	10 (55.6%)	18 (50%)
	30–44	3 (30%)	7 (70%)	10 (27.8%)
	45–59	1 (25%)	3 (75%)	4 (11.1%)
	≥60	0	1 (100%)	1 (2.8%)
Residence	Urban	4 (40%)	6 (60%)	10 (27.8%)
	Rural	8 (30.8%)	18 (69.2%)	26 (72.2%)
Marital Status	Single	7 (58.3%)	5 (41.7%)	12 (36.4%)
	Married	3 (21.4%)	11 (78.6%)	14 (42.4%)
	Divorced	2 (33.3%)	4 (66.7%)	6 (18.2%)
	Widowed	0	1 (100%)	1 (3%)
Sample type	Lymph node	11 (39.3%)	17 (60.7%)	28 (77.8%)
	Pleural fluid	1 (100%)	0	1 (2.8%)
	Genitourinary tract	0	1 (100%)	1 (2.8%)
	Cold abscess	0	4 (100%)	4 (11%)
	Skin	0	2 (100%)	2 (5.6%)

### Factors associated with extra pulmonary tuberculosis infection

In the current study, socio-demographic characteristics such as sex, residence, monthly income, number of individual within a room, and the absence of windows in a living room were not significantly associated with extrapulmonary tuberculosis infection. Adult patients were four times more likely to develop extrapulmonary tuberculosis disease than the pediatric age groups (COR = 3.778, 95%CI 1.070–13.333, P-0.039). In the Bivariate model, patients having previous history of contact with known pulmonary TB cases had four times more likely to develop EPTB than those patients who do not had contact with TB patients (COR = 4.105, 95%CI 1.826–9.229, P-0.001). On the other hand, HIV positive patients were found seven times more likely to develop EPTB infection than HIV negative once (CRO = 6.571, 95%CI 2.335–18.495, P<0.001). Compared with other body sites, tuberculosis lymphadenitis were three times more likely to occur than others (COR = 2.875, 95%CI 1.201–6.884, P = 0.018).When data was analyzed by multivariate logistic regression analysis, history of contact with pulmonary tuberculosis patients (Adjusted OR = 5.872, 95%CI 1.856–18.576, P-0.003), HIV infection (AOR = 12.335, 95%CI 2.608–58.349, p-0.002) and being an adult (AOR = 9.605 95%CI 1.612–57.223, p-0.013) remains strongly associated with extrapulmonary tuberculosis infection “Table 4”.

**Table 4:** Association between socio-demographic variables, co-infection with HIV virus, and some environmental factors with that of extrapulmonary tuberculosis infection at the University of Gondar hospital.

Predictors		GeneXpert result		Crude OR (95% CI)	Adjusted OR (95%CI)	P-value
		MTB	Not MTB			
Sex	Male	20	54	0.9 (0.4–1.9)		0.7
	Female	17	50	1.0 (r٭)		
Age	0–14	3	26	1.0 (r٭)		
	≥15	34	78	3.8 (1.1–13.3)	9.6 (1.6–57.2)	0.01
Residence	Urban	10	36	1.4 (0.6–3.2)		0.4
	Rural	27	68	1.0 (r٭)		
Family size (person)	<4	21	37	1.0 (r٭)		
	4–7	9	48	0.3 (0.1-.8)		0.7
	>7	7	19	0.6 (0.2–1.7)		
Monthly income	<400	18	30	1.7 (0.6–4.5)		0.6
	400–600	6	33	0.5 (0.1–1.6)		
	601–775	4	15	0.7 (0.2–2.9)		
	>775	9	26	1.0 (r٭)		
No of rooms	One	33	71	---------		1.00
	Two	4	17	---------		1.00
	Three	0	9	----------		1.00
	Four	0	7	1.0 (r٭)		
No of windows	0	21	40	4.2 (0.4–35.9)		0.7
	1–2	15	56	2.1 (0.2–18.4)		
	>2	1	8	1.0 (r٭)		
History of TB infection	Yes	6	18	0.9 (0.3–2.5)		0.3
	No	31	86	1.0		
History of TB contact	Yes	26	38	4.1 (1.8–9.2)	5.8(1.8–18.5)	0.003
	No	11	66	1.0 (r٭)		
Chronic disease	Yes	7	13	1.6 (.6–4.5)		0.231
	No	30	91	1.0 (r٭)		
HIV status	Negative	24	92	1.0 (r٭)		
	Positive	12	7	6.6 (2.3–18.4)	12.3 (2.6–58.3)	0.002
	Unknown	1	5	0.8 (0.09–6.9)		

CRO = Crude odds ratio, AOR = Adjusted odds ratio, CI = Confidence Interval

r٭ = reference

## Discussion

It is estimated that between 10 to 25% of tuberculosis infections occur in extrapulmonary sites worldwide. Now a day’s extrapulmonary tuberculosis is becoming a major concern of tuberculosis control programs. Formerly, it was more prevalent in developed nations than developing countries but these days, it has high proportion in developing nations including Ethiopia. The current study was conducted to determine the prevalence of *Mycobacterium tuberculosis* complex isolates from different body sites among EPTB suspected cases. The overall prevalence of culture confirmed *Mycobacterium tuberculosis* complex infection among the EPTB suspected cases in Gondar was 29.8%. Previously, the incidence of EPTB infection in Ethiopia was reported as 33% [16]. The current study also showed that the prevalence of extrapulmonary tuberculosis infection among males was slightly higher (27%) than among females (25.4%). This result is different from study reports in the USA which documented a 16.9% prevalence of EPTB infection among females compared with a 9.3% among males [25]. Another study also reported that EPTB was more common among younger ages (< 25 years) and in females [26]. Extrapulmonary tuberculosis infection was more prevalent (30.4%) among adult patients than pediatric age groups in Gondar. However, previous reports documented that EPTB infection was more common at younger ages (< 25 years) [27]. In addition, reports showed that the incidence of EPTB versus PTB decreased significantly for each decade increase in patient age [28].

Patients living in rural areas had the highest prevalence of EPTB infection (73%), and about one third (34%) of them had less than birr 400 monthly income. This low income certainly exposed the patients to inadequate nutrition which favors both pulmonary and EPTB infections. Malnutrition profoundly affects cell-mediated immunity which is the principal host defense against TB [29]. In fact, malnutrition contributes to both mortality and morbidity due to tuberculosis infection [30]. Dietary depletion has a major impact on immune function and depression of the lymphocyte function is not desirable in an individual fighting invasive mycobacterial infection. In a rat model as a consequence of malnutrition, there were lower numbers of AMs in the bronchoalveolar lavage fluid [31]. Moreover, monocytes obtained from malnourished adult patients suffering from fibrocaseous TB showed inadequate stimulation even with recombinant gamma interferon [32].

In this study, tuberculosis lymphadenitis was the most common (33.3%) form of EPTB. Previously, the proportion of TB lymphadenitis cases was reported as 82.4% in the same area [24]. According to literatures, a proportion of as high as 67% TB lymphadenitis was reported in Ethiopia [33]. A report from Tanzania showed pleural effusion being the commonest site for extrapulmonary tuberculosis infection [34].On the other hand, genitourinary tract cases were the dominant site of infection for EPTB in Hong Kong, which accounted for one-third of the total cases [11]. As reported earlier, lymph nodes and the genitourinary system were the most affected sites, followed by bones and joints, intestines, and the peritoneum [35]. Reports also showed that the frequency distribution of EPTB infection did not vary markedly between studies in Europe and the Americas, with the exception of genitourinary TB which was detected in only two of the five studies conducted in the Americas [36] as opposed to 8 of the 10 studies conducted in Europe [37].These differences might be due to the dynamics of EPTB epidemiology specific to geographic location and population. Note that the Ethiopian human population is characterized by a high level of genetic diversity [38].

Compared to previous reports [24, 39] in the same area and Addis Ababa (9.9% and 15.9%, respectively), the current prevalence of *Mycobacterium tuberculosis* complex caused EPTB (29.8%) is higher. In the Southern Region of Ethiopia, a 28% prevalence of *Mycobacterium tuberculosis* complex infection was reported [40]. Moreover, as high as a 39.7% prevalence of *Mycobacterium tuberculosis* complex caused extrapulmonary tuberculosis infection was reported in Ethiopia [41]. In Maltaya/Turkey, a prevalence of 25.9% was reported earlier [42] while prevalence of 5% was detected in Nigeria [43]. Understanding the reasons for the difference in EPTB infection prevalence could be difficult although several reports showed that HIV infection is the commonest risk factor for EPTB infection. Moreover, EPTB prevalence variation was reported to depend on sex, age group, and HIV status [44, 45]. The two most important risk factors that were significantly associated with extrapulmonary tuberculosis infection in the present study were HIV infection and history of contact with known pulmonary tuberculosis patients (P< 0.003). Different previous reports showed that being female, HIV infection, contact history with pulmonary TB cases, previous history of TB infection, and low income were documented as independent risk factors for EPTB infection [46, 47]. Tuberculosis and HIV/AIDS have a high synergistic effect on each other that one increases the progress of the other. Many scholars indicate that the presence of HIV infection increases the magnitude of EPTB infection. The immune deficiency syndrome due to the virus confers the dissemination of the bacilli from the primary site of infection, the lung, to other body parts. This is because of low or no granuloma formation at the time of TB-HIV co-infection and functional disruption of the local immune response within the granuloma [48]. In this study, the HIV status of EPTB suspected patients was determined by rapid HIV test method. All KHB positive samples were re-tested with STAT-PACK for confirmation and there were no discordant results. The study was conducted between March-April 2015 prior the KHB HIV test removed from the USAID List of Approved HIV/AIDS Rapid Test Kits as of 28^th^ July 2015.

In the current study, history of contact with known pulmonary tuberculosis patients had a significant association with EPTB development, but history of previous TB infection was not a significant risk factor. This might be due to the fact that all patients in this study who had history of pulmonary TB infection completed the full dose of anti-tuberculosis treatment and were cured. In the current study, the prevalence of HIV infection among *Mycobacterium tuberculosis* complex confirmed cases was 63.2%.

The result of cytology, GeneXpert, and culture investigations showed a prevalence of 53.2%, 26.2% and 29.8%, respectively. Discordant test result agreement was observed among cytology, GeneXpert, and culture. Taking culture as the gold standard, the sensitivity of GeneXpert assay was found to be 87.5% and the specificity of cytology examination 75%. Previously, the sensitivity and specificity of the GeneXpert method to detect *M*. *tuberculosis* complex from fine needle aspirates of tuberculosis lymphadenitis cases in Jimma, Southwestern Ethiopia, was reported as 87.8% and 91.1%, respectively. By the same study, cytology showed the lowest specificity (57.8%) [49]. In Tunisia, the sensitivity and specificity of the GeneXpert assay were reported as 87.5% and 73.3%, respectively [50]. In a country with a limited laboratory capacity like Ethiopia and high EPTB infection, the diagnosis of extrapulmonary tuberculosis is established when clinical presentation and cytological microscopic features from EPTB samples are strongly suggestive of tuberculosis [51]. Cytology is recommended as the initial diagnostic test in a TB suspected accessible mass lesion as the cytology criterion of TB has been well- established. However, it is often difficult to distinguish tuberculosis lesions from other granulomatous conditions, non-tuberculosis mycobacteria, and atypical lesions in advanced HIV disease on cytology. Similarly, doubtful cases arise on routine examination of body fluid because of poor specificity of cytology and biochemical markers, which lead to a difficult differentiation of lymphocyte-predominant “tuberculosis” cytology pattern from non-tuberculosis lesions [52]. Culture methods are much more sensitive because fewer bacilli (10–100 bacilli/ml of concentrated material) can be detected providing the necessary isolates for conventional drug susceptibility test, and species identification [53].

In the present study, only one sample demonstrated MDR *Mycobacterium tuberculosis* complex infection among the culture positive isolates making the prevalence of MDR-TB 2.4%. The prevalence of MDR-TB infection was reported as 1.3% from TB lymphadenitis cases in major towns of the Amhara Region, including the study area. Another report from Addis Ababa showed a 2.3% MDR-TB among newly diagnosed EPTB cases [54, 55]. In the northwestern Ethiopia, the prevalence of MDR-TB among EPTB cases was found to be 3.7% [55].

The limitation of this study was that samples, like cerebrospinal fluids were not included for which the prevalence of tuberculosis meningitis was not determined. In addition, AFB staining was not conducted on any of the samples.

## Conclusion

The prevalence of culture confirmed *Mycobacterium tuberculosis* complex infection in Gondar was higher. The most prevalent type of extrapulmonary tuberculosis infection was TB lymphadenitis, and adults were more at risk of developing EPTB than the pediatric group. A significant proportion of EPTB cases were also co-infected with HIV. Patients who had contact with active pulmonary tuberculosis infections had a higher chance of getting extrapulmonary tuberculosis infection. There was a significant difference between cytology results compared with GeneXpert and culture results. Extrapulmonary tuberculosis case detection strategies in Ethiopia need to be standardized. A more accurate test would contribute to improve EPTB case-detection, and thus reducing the morbidity and mortality.
